# A Review of Epidemiological Distribution of Different Types of Fractures in Paediatric Age

**DOI:** 10.7759/cureus.1624

**Published:** 2017-08-28

**Authors:** MN Baig

**Affiliations:** 1 Orthopaedics, Galway University Hospital

**Keywords:** paediatrics, buckle fracture, supracondylar fracture

## Abstract

Introduction

Treating paediatric patient fractures comprises a large part of any orthopaedic trauma service. The majority of fractures take place during sports and recreational activities. In this study, we examined the incidence of fractures and their distribution according to patient age.

Methods

We collected retrospective data from all the paediatric age group patients (under age 18) referred to our orthopaedic service from August 2015 to July 2016. We collected data for 1022 patients during one calendar year.

Results

We noted 1022 paediatric fracture presentations in one calendar year, with a 48.63% incidence in male patients and 51.36% in female patients. The age with the highest incidence was 16 years in boys and 11 years in girls. Upper limb fractures were more common than lower limb fractures in most of the subgroups.

Conclusions

These insights into paediatric fracture distribution provide an opportunity to evaluate the resources in hospitals allocated to emergency and orthopaedic departments regarding their capacity to treat fractures in paediatric patients.

## Introduction

Paediatric injuries comprise a large subset of emergency and orthopaedic outpatient presentations. The high number of paediatric injuries and fractures can be attributed to the enthusiasm typically seen in paediatric patients as they discover and experience new things while remaining unaware of the consequences. Bone properties of patients in this age group will also influence the incidence and pattern of fracture. Paediatric fractures constitute approximately 25% of all paediatric age group injuries [1]. The majority of fractures in paediatric patients are not life-threatening and are treatable [2]. Although there are many systemic and metabolic diseases that can cause or contribute to the fractures, the majority of these fractures are secondary to trauma [3].The aim of our study is to look at the incidence of fractures in paediatric patients, the prevalence of the different types of fractures, their gender distribution, and their relationship to certain activities or sports.

## Materials and methods

Our study is based on a retrospective collection of data of paediatric patients who presented with fractures to the orthopaedic outpatient services of University Hospital Kerry, as well as patients admitted for inpatient treatment. Patients under 18 years of age who presented to the University Hospital Kerry from August 2015 to July 2016 with fractures were included in the study. The epidemiological data was collected from the hospital and departmental medical records. All X-rays were reviewed to ensure that non-fracture or soft-tissue injuries were excluded. The data was collected on Microsoft Excel sheets and analysed using the Statistical Package for the Social Sciences (SPSS (version 19.0) IBM, New York, USA).

## Results

The total population of County Kerry (located in Southwest Ireland) is 147,554. The paediatric population (those under 18 years of age) is 34,940, comprising 24.013% of the total population. A total of 1022 paediatric patients presented with fractures to the orthopaedic outpatient department, making the incidence rate 29.23 fractures/1000/year. The detailed results of the epidemiological distribution of fractures are shown in Table 1.

**Table 1:** Common fractures and their epidemiological distribution according to age, sex, and frequency of different types of fractures

Fracture	Frequency	Percent	Age (year)	Sex (Male:Female)
Clavicle	53	5.2	9.21	74:26
Proximal humerus	18	1.8	11.56	44:56
Distal humerus/Supracondylar	142	13.9	6.89	51:49
Radius/Ulna diaphysis	36	3.5	9.42	50:50
Radius metaphysis	58	5.7	8.41	41:59
Distal radius /Buckle	278	27.2	8.48	54:46
Scaphoids	35	3.4	13.37	40:60
Metacarpals	51	5.0	14.02	47:53
Phalanx fingers	85	8.3	12.85	39:61
Tibia diaphysis	13	1.3	8.00	46:54
Distal tibia	9	0.9	5.00	67:33
Femur diaphysis	19	1.9	14.42	42:58
Proximal tibia	25	2.4	8.56	88:12
Patella	7	0.7	13.29	29:71
Ankle	94	9.2	12.36	40:60
Toe phalanx	16	1.6	12.88	44:56
Metatarsals	26	2.5	11.23	54:46
Pubic rami	3	0.3	16.00	0:100
Olecranon	11	1.1	7.27	18:82
Hook of hamate	7	0.7	14.00	57:43
Ulnar styloid	18	1.8	12.00	100:0
Radial head	18	1.8	8.33	33:67

**Figure 1:**
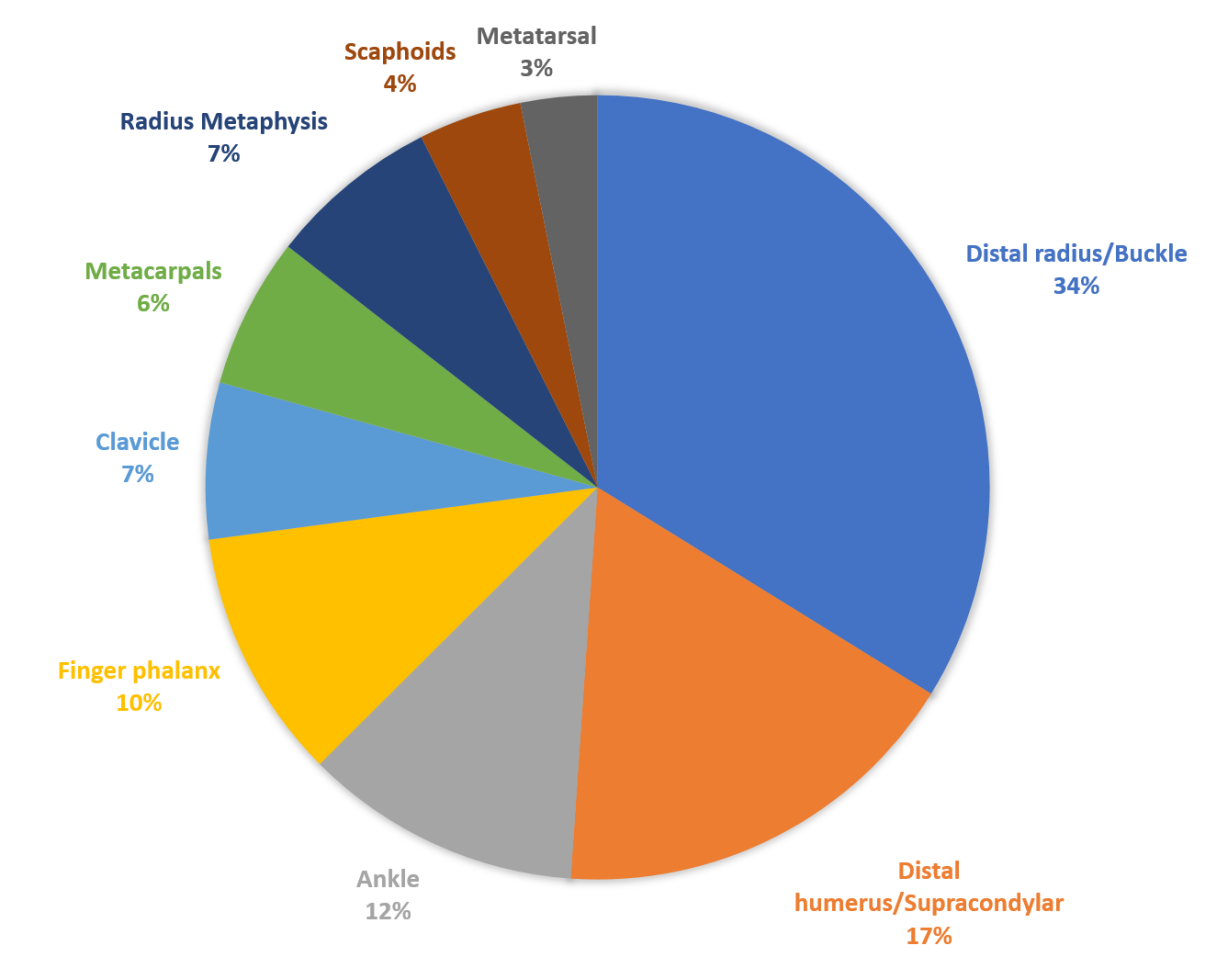
The nine most common fractures and their ratios

The most common fracture was distal radial/buckle fractures (27.2%), followed by distal humerus /supracondylar fracture (13.9%), ankle fractures (9.2%), phalanx fractures (8.3%), and radial/ulnar metaphysis fractures (5.7%). Figure 1 presents the nine most common fractures and their ratios.

The mean age of paediatric patients presenting with distal radial fracture was 8.48 years with a male/female (M/F) ratio of 54:46. The mean age of paediatric patients presenting with distal humerus fracture was 6.89 years with an M/F ratio of 51:49. The mean age of patients with ankle fractures was 12.36 years with an M/F ratio of 40:60. The mean age of patients with a phalanx fracture was 12.85 years with an M/F ratio of 39:61. Finally, the mean age of patients with a radial/ulnar metaphysis fracture was 8.41 years with an M/F ratio of 41:59. Table 2 presents fracture data according to age, M/F ratio, limb distribution, and common fractures.

**Table 2:** Paediatric subgroups according to age with sex ratio, limb distribution, and common fracture distribution

Age group (years)	Male (%):Female (%)	Upper:Lower Limb (%)	Five Most Common Fractures (%)	
0-2	33:67	100:0	Distal radius /buckle fracture	29.17
			Distal humerus/supracondylar	25
			Clavicle	25
			Ankle	12.5
3-6	50:50	81:19	Distal humerus/supracondylar	32.35
			Distal radius /buckle fracture	32.35
			Radius metaphysis	9.66
			Clavicle	7.56
			Proximal tibia	6.3
7-12	45:55	74:26	Distal radius/buckle fracture	37.28
			Distal humerus/supracondylar	11.61
			Ankle	8.93
			Phalanx fingers	8.705
			Radius/ulna diaphysis	8.036
13-17	54:46	66:34	Ankle	14.74
			Phalanx fingers	14.74
			Metacarpals	13.78
			Distal radius /buckle fracture	8.65
			Scaphoids	8.33

Up to the age of two years, the most common fractures were distal radial buckle fractures (29.17%), followed by distal humerus/supracondylar fractures in 25% of patients in this age group. Between the ages of three and six years, the most common fractures presented were distal humerus/supracondylar fractures (32.35%), distal radial/buckle fractures (32.35%), and radial/ulnar metaphysis fractures (9.66%). Between age seven and age 12, the most common fractures were distal radial/buckle fractures (37.28%), followed by supracondylar fractures (11.61%) and ankle fractures (8.93%). In patients aged 13 to 17, the most common fractures were ankle fractures (14.74%), phalanx fractures (14.74%), followed by metacarpal fractures (13.78%). Table 3 presents the fracture epidemiology according to activities.

**Table 3:** Common fractures caused during different sports/activities

Activity or Mechanism	Mean Age (years)	Male (%): Female (%)	Upper: Lower Limb (%)	Most Common Fractures (%)	
Blunt trauma	11.417	40:60	54:46	Metacarpals	25
				Phalanx fingers	12.0
				Toe phalanx	12.0
Cycling	9.889	22:78	83:17	Clavicle	33.3
				Proximal humerus	33.3
				Phalanx fingers	16.6
				Metatarsals	16.6
Fall from bed/chair	5.737	49:51	74:26	Clavicle	21.0
				Distal hum/supracondylar	20
				Proximal tibia	13.6
				Distal radius/buckle fracture	10.5
				Scaphoids	6.32
Fall on outstretched hand	7.864	66:34	98:2	Distal radius/buckle fracture	72.4
				Radius metaphysis	9.5
				Scaphoids	7.24
Gaelic Soccer	14	23:77	84:16	Distal radius/buckle fracture	17.2
				Clavicle	13.5
				Phalanx fingers	13.5
				Radius metaphysis	12.3
Hockey	13.034	76:24	90:10	Phalanx fingers	34.4
				Metacarpals	27.5
				Olecranon	10.3
				Ankle	10.3
Hurling	13.567	80:20	93:7	Phalanx fingers	20
				Distal radius/buckle fracture	20
				Proximal humerus	20
				Metacarpals	10
				Scaphoids	10
Camogie	12.667	100:0	67:33	Phalanx fingers	33.3
				Ankle	33.3
				Radius/ulna diaphysis	33.3
Rugby	14	0:100	100:0	Clavicle	66.6
				Phalanx fingers	33.3
Soccer	10.937	45:55	69:31	Distal radius/buckle fracture	35.4
				Ankle	21.7
				Radius/ulna metaphysis	10.2
Trampoline/monkey bar	7.256	46:54	95:5	Distal humerus/supracondylar	80.8
				Distal radius/buckle fracture	9.6
				Ankle	4.8

A commonly reported cause of injury was blunt trauma, occurring in 54% of the fractures of the upper limb and 46% of the fractures of the lower limb. The most common fractures due to blunt trauma were metacarpal fractures (25%), finger phalanx and foot phalanx fractures (12% each), followed by ankle fractures (11.11%). Another common mechanism was falling on an outstretched hand, and the associated fractures were distal radial fractures (72.4%), radial metaphysis fractures (9.5%), scaphoid fractures (7.24%), and radial head fractures (2.71%). Fractures associated with trampolines, monkey bars, and bouncing castles were common in younger children. The most common fracture pattern seen in these activities was distal humerus/supracondylar fractures (80.8%), distal radial/buckle fractures (9.6%), followed by ankle and phalanx fractures (4.8% each). The fracture pattern seen resulting from Gaelic football injuries were distal radial/buckle fractures (17.28%), clavicle fractures (13.58%), finger phalanx fractures (13.58%), radial/ulnar fractures (12.35%), and distal humerus/supracondylar, metacarpals, and tibia fractures (7.41% each). Fractures associated with hurling (a popular outdoor stick and ball field sport) were finger phalanx, distal radius, and proximal humerus fractures (20% each), followed by metacarpal and scaphoid fractures (10% each).

In the female population, camogie (a sport similar to hurling) and hockey are very popular activities. The most common fracture seen in camogie players were the ankle, phalanx, and radial/ulnar shaft fractures (33.3% each). For hockey players, finger phalanx fractures were most common (34.48%), followed by metacarpal fractures (27.59%), ankle (10.34%), and olecranon fractures (10.34%). Fractures associated with soccer were distal radial/buckle fractures (35.43%), ankle fractures (21.7%), and radial/ulnar metaphysis fractures (10.29%).

**Figure 2:**
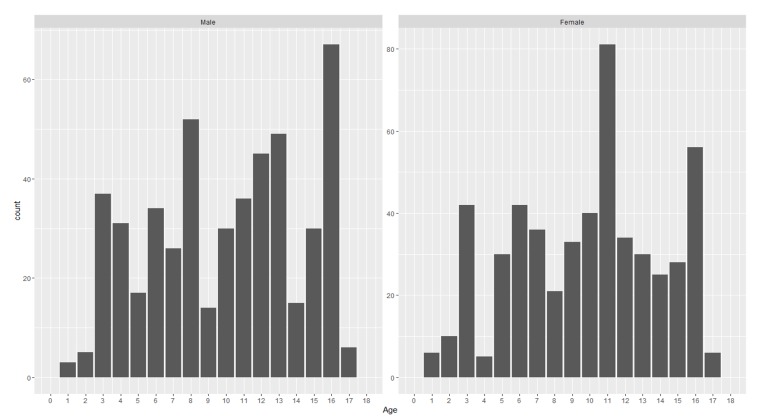
Distribution of fractures in the male/female population and fracture distribution according to age

The other common activities/mechanisms reported included cycling, road and traffic accidents (RTA), and falls from beds or chairs. See Table 3 for common fractures during those activities. The distribution of fractures in male/female population and the fracture distribution according to age is shown in Figure 2.

## Discussion

Fractures commonly occur in paediatric and elderly patients because of relatively weaker points of physis and metaphysis, and in elderly patients because of deteriorated bone quality [4]. The incidence rate of fractures in the paediatric population has ranged from 12.8/1000 as reported by Kopjar, et al. in Norway [2] to 36.1/1000 as described by Lyons, et al. [5] in Wales. The incidence rate in our study was 29.2/1000, near the higher end of the range. Many variables can affect the incidence, including the size of the paediatric population and the social emphasis on encouraging physical activity. The distribution of fractures between the upper and lower limbs has a certain pattern depending on the age. Early in life, children’s activities utilise upper limbs rather than lower limbs, but as they start walking and running, the incidence of lower limb fractures increases.

According to our findings, the distribution of upper and lower limb fractures show specific trends that are quite representative of the nature of the sports related to those fractures. The distribution of fractures among male and females is shifted more towards males in our study, which may be due to the difference in activity levels. In the literature, distal radial fractures are the most common fractures in all paediatric age groups, which aligns with our findings [6]. The incidence and pattern of the fractures differ by location due to lifestyle differences such as rural vs. urban, area topography, and social and economic parameters.

## Conclusions

This study provides an accurate assessment of the fractures in paediatric patients distributed by type, age, gender, and activity. This information can help allocate resources for dealing with these injuries in emergency and outpatient departments. These findings may also help healthcare professionals educate parents, guardians, school staff, paramedic staff, hospital staff, and the public in general, on common injuries in children and their relations to certain activities, in efforts to help minimise those injuries.
